# Effect of Debagging Time on Pigment Patterns in the Peel and Sugar and Organic Acid Contents in the Pulp of ‘Golden Delicious’ and ‘Qinguan’ Apple Fruit at Mid and Late Stages of Development

**DOI:** 10.1371/journal.pone.0165050

**Published:** 2016-10-27

**Authors:** Chenjuan Jing, Changqing Ma, Juan Zhang, Shujuan Jing, Xiaobing Jiang, Yazhou Yang, Zhengyang Zhao

**Affiliations:** 1 Department of Horticulture, Northwest A&F University, Yangling, Shaanxi, People’s Republic of China; 2 Shaanxi Research Center of Apple Engineering and Technology, Yangling, Shaanxi, People’s Republic of China; 3 Apple Experimental Farm of Northwest A&F University, Baishui, Shaanxi, People’s Republic of China; Beijing Forestry University, CHINA

## Abstract

This study examined the effect of debagging time on color and flavor / taste compounds in the non-red apple cultivar ‘Golden Delicious’ and red cultivar ‘Qinguan’ at mid and late stages of fruit development. Debagging briefly improved the red color in both cultivars, the peel of ‘Golden Delicious’ presenting pale-pink hue. However, rapid anthocyanin accumulation occurred in apple peel at a specific time (after 179 days after flowering (DAF) in ‘Qinguan’) and was unaltered by debagging time in the red cultivar ‘Qinguan’. Furthermore, untimely debagging had a detrimental effect on the content of anthocyanin. All sugars increased and organic acids decreased in apple pulp at mid to late stages of development. Bagging treatment reduced the content of most sugars and organic acids, as well as, the overall total. However, glucose and citric acid contents were higher in bagged fruit than non-bagged fruit; the maximum occurred in T7 treatment that was no-debagging at DAF 159 / 196 (‘Golden delicious’ / ‘Qinguan’), i.e., 24.35 and 0.07 mg g^-1^ FW in ‘Golden delicious’, and 38.86 and 0.06 mg g^-1^ FW in ‘Qinguan’, respectively. In a word, bagging treatment can alter the pattern of peel color development in apple fruit; however, it remains difficult to alter the timing of rapid anthocyanin accumulation as it is regulated solely by development. Moreover, bagging treatment reduced the total accumulation of sugars and organic acids, and even the over total in pulp, but increased the glucose and citric acid contents in apple pulp.

## Introduction

Commercially, the quality of apples in the marketplace is determined firstly by visual appearance-bright and clean color on the peel, and the flowing is taste which is determined mainly by the sugars and organic acids. There are three colors of apple, red, green and yellow. Different cultivars have their own unique color, thereby providing consumers with essential information on the variety and even cultivar management. In young fruit, the peel of most apple cultivars is green in color, the color changes with ripening in pigment components [1–3]. In red apples in particular, the color composition of the peel begins to change after the fruit expansion period. During cultivation, the peel color of different cultivars therefore differs, giving each their distinct final appearance and taste.

Flavor and taste compounds in apple pulp are also important commercial traits, and are governed by the ratio of total sugars to organic acids [4, 5]. In addition to the change in peel color in mid to late fruit development, flavor and taste compounds in the pulp are also modified. Metabolism of carbohydrates in fruit is complex. In general, starch is deregulated, resulting in an increase in total sugars and decrease in organic acids [6–8]. Upon reaching maturity, the starch content is substantially low, while the ratio of sugars to organic acids tends to be balanced. The taste index, represented by the ratio of sugars to organic acids, is a critical reference value for fruit quality in pomology [4, 6, 9].

Red apple cultivars are the result of anthocyanin accumulation in the peel. Anthocyanins, ubiquitous secondary metabolites, are water-soluble pigment found, widely across the plant kingdom, being responsible for the orange, red, purple and blue colors of petals, fruits, roots, tubers, stems and leaves [10–13]. Anthocyanins in apple peel are also attributed to the health attributes of mature fruit, whilst protecting plants from pathogens, herbivores and UV light [14, 15]. Anthocyanins accumulation was codetermined by genetic and environment factors. The biosynthetic pathway of anthocyanins has been studied in detail, especially in maize, Arabidopsis, snapdragon and petunia [16–18], revealing the importance of certain structural genes (*CHS*, *CHI*, *F3H*, *DFR*, *ANS* and *UFGT*) and regulation of certain factors such as the *Myb-bHLH-WD40* protein complex [19]. The environment cues were included with mainly light, temperature, pH values and so on [20, 21]. In red apples, for example, light induces expression of the *MYB* gene, which in turn regulates anthocyanin accumulation [22]. Consequently, anthocyanin biosynthesis is prevented by bagging treatment, while debagging improves the coloration of fruit peel at right time [23, 24] Carbohydrates, in particular exogenous sucrose, have also been shown to have a positive effect on anthocyanin biosynthesis in a number of plant seedlings [10, 25–28]. However, in apples, anthocyanin accumulation remains under the control of fruit development [29, 30]. In mid and late stages in particular, a number of flavor and taste compounds are synthesized simultaneously. The effect of endogenous sugars and organic acids on anthocyanin biosynthesis in apples remains unknown.

Bagging treatment is a common practice in production of fruits [23, 31]. The original usage of bagging is to protect against insect, pesticides and mechanical scratches. Now the major role of this practice at the right time was to improve the external quality of fruits [23, 24, 32]. However, the impact of bagging on the components involved in taste of fruit was still controversial in field production. This approach could reduce the substances of flavor and taste. For example, Liu et al found the contents of total sugars and total acids in‘Granny Smith’ and ‘Golden Delicious’ with bagging were lower than that without bagging fruits, and Feng et al also had the same result in ‘Jonagold’ [1, 8].Whereas, debagging treatment of apple in these studies is all at right time, they did not mention the effect of the different debagging time. The aim of this study was to assess the effect of debagging time on patterns of pigment accumulation in the peel and sugar and organic acid accumulation in the pulp of apple fruit, and determine the relationship between these compounds during mid to late fruit development under different time of debagging treatments. For this purpose, we performed a comparative study concerning peel color and sugar and organic acid contents in apple fruit between the non-red cultivar ‘Golden Delicious’ and deep red cultivar ‘Qinguan’.

## Materials and Methods

### Study site and plant materials

Trees of ‘Golden Delicious’ (non-red cultivar) and ‘Qinguan’ (deep-red cultivar) (*Malus × domestica* Borkh.) were procured from an apple experimental station of Northwest A&F University (Yangling, China). The station lies within the continental monsoon zone (109°32′50″ E, 35°12′25″ N) in Baishui County, Shaanxi Province, China. The elevation is 830 m, with an average annual temperature of 11.6°C and average annual rainfall of 567.6 mm. All cultivars were grafted onto M26 rootstocks (*Malus× domestica* Borkh.). Trees aged 8–10 years were selected and planted at a density of 3 m × 1.2 m, each cultivar had three replicates of nine trees, and each tree had all treatments. What’ more, nine fruits of each treatment were collected from the nine trees, respectively.

### Treatments and sampling

Juvenile fruit was covered with light-impermeable two-layer paper bags at approximately 45 days after flowering (DAF). Bags consisted of a yellow outer and red waxy inner layer and were 14 cm × 16 cm. Bags were removed and samples harvested in mid to late stages of fruit development: ‘Golden Delicious’ at 90 (T1), 108 (T2), 122 (T3), 138 (T4), 145 (T5), 152 (T6), and 160 (T7) DAF, and ‘Qinguan’ at 126 (T1), 137 (T2), 152 (T3), 168 (T4), 179 (T5), 187 (T6), and 196 (T7) DAF. Each time point had a separate debagging treatment and fruit samples were first harvested at the time of bag removal. These early treatments were then harvested at the next time points until the last time for observing fruit development. We also collected no-bagged samples (CK) of per cultivar for the blank control at the same time (S1 Table). All samples were harvested between 17:00–18:00. Before destroying the fruit, the redness of the peel was determined. Then we collected the peel and pulp of every fruit, respectively. The peel was collected three rings around every fruit equator; and the pulp was collected at the symmetrical positions of a fruit. They were immediately immersed in liquid nitrogen and maintained at –80°C until the analysis was performed.

### Measurements of peel color, chlorophyll and carotenoids

Peel color was measured using a colorimeter (CHROMA METER CR-400 Chroma Portable, Konica Minolta Sensing, Inc., Osaka, Japan). Fruit color is recorded using CIE parameters L*, a* and b* [33, 34]. Here, we focused on peel redness using values of a*, a negative value representing green and positive red. Color measurements were taken the average of five points around the equator per fruit.

Chlorophyll and carotenoid pigments were extracted using 80% chilled acelone and detected using an ultraviolet spectrophotometer (UV-2550) [35].

### Quantification of anthocyanin, sugars and organic acids

Anthocyanin was measured using the protocol of Liu et al. (2013). Frozen peel samples (1.00 mg) were homogenized in 5 ml 0.25% (v/v) HCl: MeOH (0.25: 99.75, v/v) at 4°C for 24 hours on a rotating wheel in darkness. After centrifugation at 3,500 g for 10 min, the supernatant was transferred to autosampler vials for high-performance liquid chromatography (HPLC) analysis. Abundant cyanidin 3-O-galactoside (cya 3-gal) and trace amounts of cyanidin 3-O-glucoside (cya 3-glu) are observed in apple peel, and were therefore used as standards (Sigma Chemical, St. Louis, MO, USA). HPLC parameters were as follows: sample size, 5μL; overall flow rate, 1 mL·min^−1^; C 18 column (5 μ, 250 × 4.6 mm internal diameter, Diamonsil, China) maintained at 40°C; separation followed by testing of samples using a photodiode array detector (Waters 2998) at 530 nm [36].

Sugar and organic acid contents were determined as described by Liu et al. (2012) [1].

### Statistical analysis

All data from at least three replicates per treatment were analyzed using SPSS Statistics (Version 17.0, SPSS Inc., Chicago, IL, USA). Means were analyzed using one-way analysis of variance. Multiple comparisons among all treatments’ dates of all time points were conducted using Tukey’s test at *P* < 0.05.

## Results

### Effect of debagging time on the color parameter a* values

**Fig 1:**
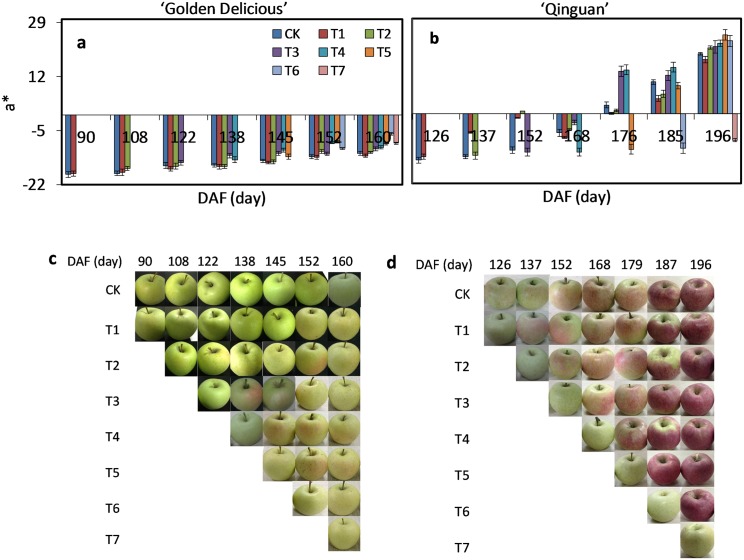
Dynamic characteristics of the color parameter a* in peel of ‘Golden Delicious’ and ‘Qinguan’. Fruit was collected from 90 to 160 days after flowering (DAF) (‘Golden Delicious’) and 126 to 196 DAF (‘Qinguan’). CK, no-bagging; T1, debagging at 90/126 DAF (‘Golden Delicious’ / ‘Qinguan’); T2, debagging at 108/137 DAF; T3, debagging at 122/152 DAF; T4, debagging at 138/168 DAF; T5, debagging at 145/179 DAF; T6, debagging at 152/187 DAF; and T7, debagging at 160/196 DAF. Bars indicate ± standard error of three independent replicates. Significant differences among treatments at each sample date were determined according to Tukey’s test, *P <* 0.05.

As a measurement of peel color, red or green, a* values were used. In ‘Golden Delicious’, a* remained negative in all treatments, with only a slight increase with the process in development (Fig 1a), while a* values for CK (with no-bagging) were 2%–36% higher than with bagging treatment. Peel of ‘Golden Delicious’ was therefore predominantly non-red.

In ‘Qinguan’, almost consistently negative a* values were observed from 126 to 176 DAF, regardless of debagging time (Fig 1b). Thereafter, a gradual increase was observed until all values were positive. Furthermore, the earlier the debagging treatment was conducted, the lower the a* values were. A minimum a* value of 17.07 was observed with T1 at 196 DAF and a maximum of 24.95 with T5 at 179 DAF. Peel of ‘Qinguan’ therefore showed a gradual change from green to light red then from cardinal (before about 168 DAF) to even deep red (from about 168 DAF to maturity). Because the first samples of each debagging treatment did not remove bags, a* values of these samples did not obviously change for both ‘Golden Delicious’ and ‘Qinguan’; they were all negative and increased slightly (S2 Table).

### Accumulation of chlorophyll, carotenoid and anthocyanin

**Fig 2:**
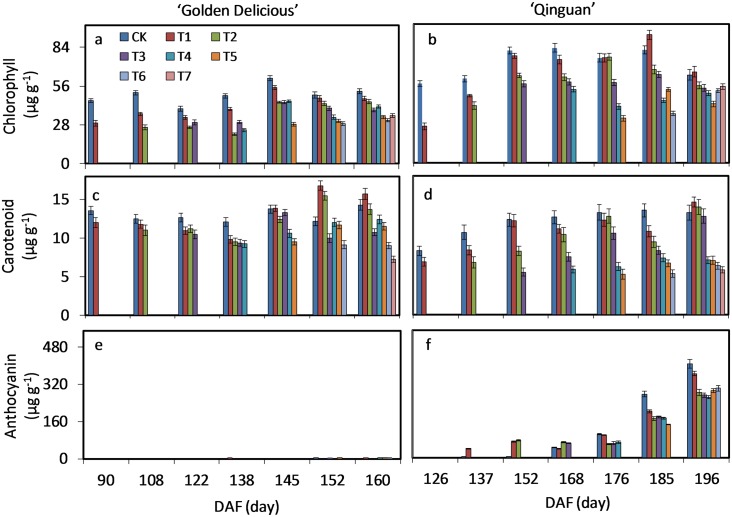
Accumulation of chlorophyll, carotenoid and anthocyanin in peel of ‘Golden Delicious’ and ‘Qinguan’. The harvesting and debagging time wrer shown in Fig 1. Bars indicate means ± standard error of three replicates. Significant differences among treatments at each sample date were determined according to Tukey’s test, *P <* 0.05.

The color of apple peel is determined by the composition of carotenoid, chlorophyll (non-red cultivars) and anthocyanin (red cultivars). Carotenoid and chlorophyll represent the ground color components, and anthocyanin represents the over color. Contents of carotenoid and chlorophyll were consistently higher with no-bagging (CK) in both cultivars than that with bagging treatments. In mature fruit, contents of carotenoid and chlorophyll of CK were 52.2 and 14.29 μg mg^-1^ in ‘Golden Delicious’ and 65.51 and 15.71 μg mg^-1^ in ‘Qinguan’, respectively. Contents with no-bagging were consistently higher in ‘Qinguan’ than ‘Golden Delicious’ (Fig 2a–2d). These findings suggest that the base colors of green and yellow did not significantly change with no-bagging, instead only fluctuation within a definitive range in both cultivars. Compared with bagging treatment (T7), there were more chlorophyll (8–43%) and carotenoid (9–200%) to accumulate in apple peel of mature fruit with debagging treatment; however, they never exceeded the contents with no-bagging (CK) (S3 Table).

Anthocyanin in the peel of ‘Golden Delicious’ with debagging treatment, was 0 until before about 145 DAF; however, trace amounts of anthocyanin synthesis were observed at T4, T5 and T6 (Fig 2e). In ‘Qinguan’, trace amounts of anthocyanin were observed with no-bagging up to about 152 DAF. T2 and T3 resulted in a 1.5-fold increase up to about 168 DAF, and these values were maintained up to about 185 DAF, after which they sharply increased. Furthermore, in mature fruit of ‘Qinguan’ (up to about 196 DAF), anthocyanin content with no-bagging was 1.55-fold higher than with all bagging treatments.

### Kinetics of sugar and organic acid accumulation

In apple fruit, the major sugars are fructose, sucrose, glucose and small amounts of sorbitol. These sugar contents all increased overall in apple pulp during fruit development, and the increasing changes were unaffected by debagging time in both cultivars (S4 Table). Fructose was the most abundant of all sugars, also showing the highest content with no-bagging and with early compared to late debagging treatment. Fructose of CK and T1 were 74.37 and 69.81 mg g^-1^, respectively, while at T7 the content was 64.82 mg g^-1^ in ‘Golden delicious’ at maturity.

Sucrose and glucose were the second most abundant sugars. The content of sucrose was highest with no-bagging, while with debagging treatment, the later the debagging time the lower the content at maturity. CK, T1 and T7 were 26.50, 24.21, and 16.79 mg g^-1^, respectively in ‘Golden delicious’. Glucose content showed a reverse trend. When the fruit was mature, the highest glucose content was T7 with 24.35 mg g^-1^ in ‘Golden delicious’.

**Fig 3:**
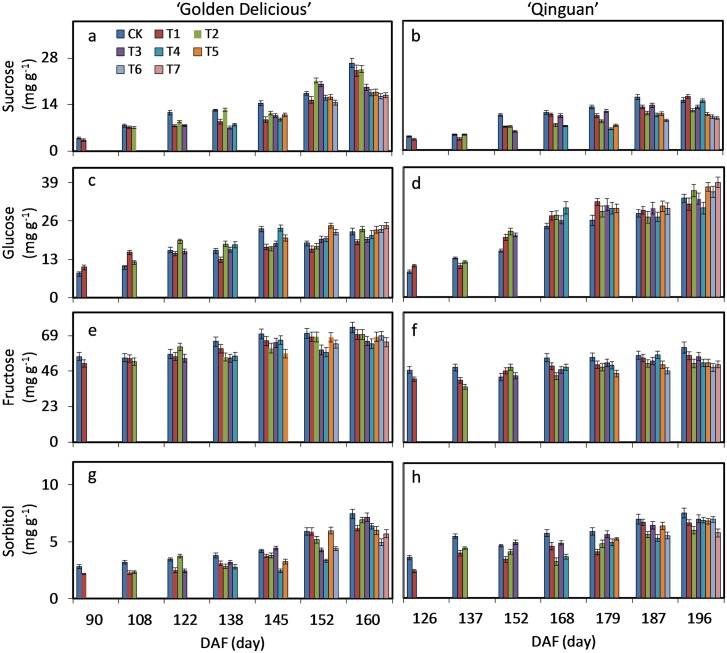
Kinetics of sucrose, glucose, fructose and sorbitol accumulation in pulp of ‘Golden Delicious’ and ‘Qinguan’. The harvesting times and treatments are as described in Fig 1. Bars indicate ± standard error of three replicates. Significant differences among treatments at each sample date were determined according to Tukey’s test, *P <* 0.05.

Sorbitol, as the main form of transport in plants, showed similar changes as sucrose and fructose with debagging treatment. CK was the highest, and T7 was the lowest in fruit at maturity. Although fructose, sucrose glucose and sorbitol showed similar trends across mid to late stages of fruit development, increasing respectively, bagging treatments reduced the contents of sugars except glucose in ‘Golden delicious’ (S4 Table). The changes of these sugar contents in ‘Qinguan’ were consistent with those in ‘Golden delicious’ (Fig 3).

In both cultivars, the most abundant organic acid was malic acid, which represented above 90% of total organic acids. Citric acid and succinic acid came second and third, respectively. These three organic acids decreased gently over time at the mid to late stages of fruit development. Except for malic acid, the remaining two organic acids seemed lower with bagging treatment (S5 Table).

Malic acid content decreased with a delay in debagging time. The content with no-bagging treatment (CK) peaked in mature fruit, with the minimum values being found in T7. CK and T7 were 9.17 and 8.29 mg g^-1^ in ‘Golden delicious’, and 6.01 and 5.09 mg g^-1^ in ‘Qinguan’, respectively.

**Fig 4:**
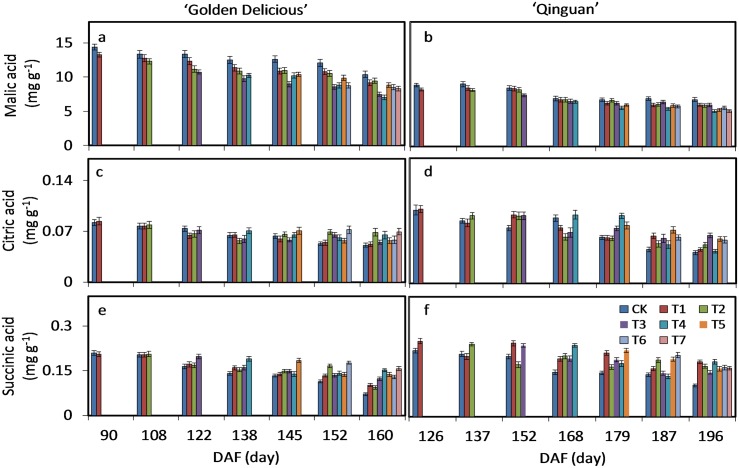
Malic acid, citric acid and succinic acid accumulation in pulp of ‘Golden Delicious’ and ‘Qinguan’. The harvesting times and treatments were as described in Fig 1. Bars indicate ± standard error of three replicates. Significant differences among treatments at each sample date were determined according to Tukey’s test, *P <* 0.05.

Citric acid content with no-debagging was almost always the highest at each collection time. T7 and CK had the citric acid content of 0.05 and 0.04 mg g^-1^ in ‘Golden delicious’, and 0.06 and 0.04 mg g^-1^ in ‘Qinguan’, respectively, in mature fruit. CK had the succinic acid content of 0.11 and 0.09 mg g^-1^ in ‘Golden Delicious’, and 0.16 and 0.07 mg g^-1^ in ‘Qinguan’, respectively. Furthermore, the changes in organic acid contents in ‘Golden Delicious’ were similar to those in ‘Qinguan’, showing a consistent declining trend (Fig 4).

## Discussion

Bagging treatment is a common practice in apple production, with the time of debagging being an empirical value. Most experiments carried out at the empirical time were concerned with finding quality differences between bagging and no-bagging [2, 8, 36, 37]. Here, we detected an effect of different debagging time treatments on apple quality in the non-red cultivar ‘Golden Delicious’ and red cultivar ‘Qinguan’. Although debagging treatment improved redness momentarily at a mid-stage of fruit development, an early debagging time had no effect.

### Effect of debagging time on peel color of apple fruit

After fruit expansion, cells almost stop growing, and peel color starts to change, representing a second peak in anthocyanin accumulation [29]. Debagging treatment was therefore started at this stage. Bagging treatment decreased the levels of chlorophyll and carotenoid in the peel of both ‘Golden Delicious’ and ‘Qinguan’ fruit, giving a lighter ground color. Moreover, the earlier the bag removal time, the higher the content. If we suppose that the non-red cultivar ‘Golden Delicious’ has only ground color pigments, barely any cover color, the ground colors in both the non-red cultivar ‘Golden Delicious’ and red cultivar ‘Qinguan’ showed the same tendency. It is hard to produce a red color in ‘Golden Delicious’, but it can occur under some conditions. In the peel of ‘Golden Delicious’, a higher content of anthocyanin was observed only when debagging was carried out at about 145 DAF, leading to the peel presenting pale-pink. Therefore, in the non-red cultivar ‘Golden Delicious’, bag removal is useful only at a specific time point, with the anthocyanin content being substantially low (Fig 2e).

During the study period from mid to late development, the color of the red cultivar ‘Qinguan’ showed marked differences with stage and bag removal time, respectively. Peel redness of all bagged fruits showed a similar trend as ‘Golden Delicious’; that is, non-red peel. Fig 2b also shows that there was no anthocyanin synthesis in bagged fruit. Light is therefore necessary for anthocyanin biosynthesis in the peel of ‘Golden Delicious’ and ‘Qinguan’ fruit. In ‘Jonagold’, ‘Red Delicious’, ‘Royal Gala’ and ‘Delicious’, anthocyanin accumulation was also found to require light [8, 38, 39]. It is therefore tempting to speculate that light is required for anthocyanin biosynthesis in most cultivars of apple.

Under the regulation of fruit development, some red cultivars experience two peaks in anthocyanin biosynthesis in the peel. The first occurs in young fruit and the second at maturity [29, 40]. In this article, we focused on the quality of fruits at mid to late stages of development. The second peak was therefore only observed after about 176 DAF, and lasted for about 25 days. Before 185 DAF, there were five separate bag removal treatments; however, rapid anthocyanin accumulation did not occur as a result of lengthening of the days of exposure. These phenomena strongly suggest that this process is mainly controlled by fruit development. When fruit stopped expanding, although the time of debagging was early, the amount of anthocyanin accumulation was increased gently, instead the content was more closely related to that of no-bagging. With debagging at 176 and 185 DAF, the content rose quickly, and this change was consistent with no-bagging treatment.

Studies of anthocyanin in the peel of mature apples mostly focus on the period of rapid accumulation, especially with bagging treatment [1, 8, 39]. Saure et al. proposed that light is required for anthocyanin accumulation in apple cultivars, with the process of phytochrome mediation probably requiring photosynthetic activity [41]. Bag removal treatment leads to light stimulation of a number of substances including structural and regulator genes of anthocyanin biosynthesis, rapidly reddening the color of the peel and causing a sharp increase in anthocyanin content [1, 42]. In other rosaceous plants, for example, peach and pear, the effect of bagging and bag removal on fruit peel is the same [23, 32].

Color variation therefore depends on genotype as well as developmental and environmental cues [43–45]. Genotypes and the features of anthocyanin development are critical and are unchanged by debagging treatment; however, bagging and debagging at optimal time could improve apple peel color. That is, it is difficult to disrupt anthocyanin biosynthesis [8, 36]. Furthermore, bagging / debagging practices were effective under the right timing in ‘Qinguan’, suggesting that the genes affecting anthocyanin biosynthesis are controlled firstly by fruit development then environment cues.

### Effect of bagging treatment on sugars and organic acids in apple pulp

In apple fruit, sugars are composed mainly of fructose, glucose, sucrose and sorbitol, and organic acids of malic acid, citric acid and succinic acid [46, 47]. With fruit development, the content of total sugars increases while that of total acids decreases [1, 8]. In the pulp of ‘Golden Delicious’ and ‘Qinguan’, these major sugars and organic acids almost consistently followed these trends, with no effect of bag removal time. That is, quantitative changes in sugars and organic acids are mainly controlled by fruit development. However, bagging and bag removal time had an effect on the amount of sugars and organic acids in apple pulp. Bagging treatment generally decreased the total accumulation of sugars and organic acids, but increased the glucose and citric acid contents in ‘Golden Delicious’ and ‘Qinguan’. Similar results were reported in the pulp of ‘Jonagold’ apples [8]. With bagging, the fruit is insulated from the environment, minimum temperatures, light and humidity all changing within the bags. The increase in contents of glucose and citric acid are therefore possibly a response of apple fruit to this environmental variation.

Sugars are an important prime carbon and energy source, performing important regulatory functions, controlling metabolism, stress resistance, growth, and development in numerous plants [46, 48, 49]. If all sugars decrease when fruits are bagged, it is possible that the normal development of fruits is also being disturbed. Glucose, a reducing monosaccharide, is the second largest sugar component in apples, acting as a reserve substance. In plant cells, glucose performs different bioactive functions. Glucose also plays major roles in metabolism including the processes of photosynthesis and respiration [50], as well as regulating a number of growth and developmental stages [50, 51]. Glucose-6-phosphate levels were found to be important in carrying out down-regulated carbon-consuming processes and normal fruit development [48, 52]. Thus, glucose consumption is elevated, enhancing the demand in bagging fruit. In fruit, sugar metabolism is a complex process. Glucose is not only a reserve material, but also an intermediate metabolite in glycolysis and respiration pathways [28, 50]. The relationship between glucose and bagging or light / temperature remains largely unknown in apple fruit.

In plants, citric acid is a metabolic product, found widely in fruit where it acts as a storage material [9, 53–55]. Citric acid is not only a naturally occurring organic acid, but is also important in that it does not destroy the taste or flavor of fruit. Citric acid can prevent browning and maintain the quality of fresh-cut samples, while exogenous citric acid can control microbial contamination of fresh-cut fruit and vegetables [54, 56, 57]. It is therefore often used as a food preservative. Our findings suggest that the relationship between bagging treatment and citric acid is reverse. In fact, bagging treatment acts to reduce light and high temperature stress, with citric acid acting as a natural antioxidant and anti-browning agent protecting the structure and metabolic reactions. As a result, citric acid helps maintain fruit development in a dark environment. The detailed roles of citric acid in bagged fruit therefore warrant further analysis in the future.

## Supporting Information

S1 Table The treatments and harvesting time of both ‘Golden delicious’ and ‘Qinguan’ apple fruit. B-baging, DB-debagging, H-harvesting.
(DOCX)

S2 Table The statistical results shows significant differences in a* values’ of ‘Golden delicious’ and ‘Qinguan’. Fruit was collected from 90 to 160 days after flowering (DAF) (‘Golden Delicious’) and 126 to 196 DAF (‘Qinguan’). CK, no-bagging; T1, debagging at 90/126 DAF (‘Golden Delicious’ / ‘Qinguan’); T2, debagging at 108/137 DAF; T3, debagging at 122/152 DAF; T4, debagging at 138/168 DAF; T5, debagging at 145/179 DAF; T6, debagging at 152/187 DAF; and T7, debagging at 160/196 DAF. Lowercase letters indicate statistically significant differences among treatments at all sample date with 9 independent replicates (*P <* 0.05).
(DOCX)

S3 Table The statistical results shows significant differences in chlorophyll, carotenoid and anthocyanin of ‘Golden delicious’ and ‘Qinguan’. The harvesting and debagging time were shown in S1 Table. Lowercase letters indicate statistically significant differences among treatments at all sample date with 3 replicates (*P <* 0.05).
(DOCX)

S4 Table The statistical results shows significant differences in sucrose, glucose, fructose and sorbitol content of ‘Golden delicious’ and ‘Qinguan’. The harvesting and debagging time were shown in S1 Table. Lowercase letters indicate statistically significant differences among treatments at all sample date with 3 replicates (*P <* 0.05).
(DOCX)

S5 Table The statistical results shows significant differences in Malic acid, citric acid and succinic acid of ‘Golden delicious’ and ‘Qinguan’. The harvesting and debagging time were shown in S1 Table. Lowercase letters indicate statistically significant differences among treatments at all sample date with 3 replicates (*P <* 0.05).
(DOCX)

## References

[1] Liu Y, Zhang X, Zhao Z. Effects of fruit bagging on anthocyanins, sugars, organic acids, and color properties of ‘Granny Smith’ and ‘Golden Delicious’ during fruit maturation. European Food Research and Technology. 2012;236(2):329–39. 10.1007/s00217-012-1896-3

[2] Sun S, Xin L, Gao H, Wang J, Li P. Response of phenolic compounds in ‘Golden Delicious’ and ‘Red Delicious’ apples peel to fruit bagging and subsequent sunlight re-exposure. Scientia Horticulturae. 2014;168:161–7. 10.1016/j.scienta.2014.01.031

[3] Xie L, Wang Z, Cheng X, Gao J, Zhang Z, Wang L. 5-Aminolevulinic acid promotes anthocyanin accumulation in Fuji apples. Plant Growth Regulation. 2013;69(3):295–303. 10.1007/s10725-012-9772-5

[4] Zhang Y, Li P, Cheng L. Developmental changes of carbohydrates, organic acids, amino acids, and phenolic compounds in ‘Honeycrisp’ apple flesh. Food Chemistry. 2010;123(4):1013–8. 10.1016/j.foodchem.2010.05.053

[5] Hecke K, Herbinger K, Veberic R, Trobec M, Toplak H, Stampar F, et al Sugar-, acid- and phenol contents in apple cultivars from organic and integrated fruit cultivation. Eur J Clin Nutr. 2006;60(9):1136–40. 10.1038/sj.ejcn.1602430 . 16670694

[6] Ma B, Chen J, Zheng H, Fang T, Ogutu C, Li S, et al Comparative assessment of sugar and malic acid composition in cultivated and wild apples. Food Chemistry. 2015;172(0):86–91. 10.1016/j.foodchem.2014.09.032 25442527

[7] Wu J, Gao H, Zhao L, Liao X, Chen F, Wang Z, et al Chemical compositional characterization of some apple cultivars. Food Chemistry. 2007;103(1):88–93. 10.1016/j.foodchem.2006.07.030

[8] Feng F, Li M, Ma F, Cheng L. The effects of bagging and debagging on external fruit quality, metabolites, and the expression of anthocyanin biosynthetic genes in ‘Jonagold’ apple (Malus domestica Borkh.). Scientia Horticulturae. 2014;165:123–31. 10.1016/j.scienta.2013.11.008

[9] Zhao J, Li H, Xi W, An W, Niu L, Cao Y, et al Changes in sugars and organic acids in wolfberry (Lycium barbarum L.) fruit during development and maturation. Food Chemistry. 2015;173(0):718–24. 10.1016/j.foodchem.2014.10.082 25466081

[10] Zhang KM, Li Z, Li Y, Li YH, Kong DZ, Wu RH. Carbohydrate accumulation may be the proximate trigger of anthocyanin biosynthesis under autumn conditions in Begonia semperflorens. Plant biology. 2013;15(6):991–1000. 10.1111/j.1438-8677.2012.00721.x . 23578316

[11] Teng S, Keurentjes J, Bentsink L, Koornneef M, Smeekens S. Sucrose-specific induction of anthocyanin biosynthesis in Arabidopsis requires the *MYB75/PAP1* gene. Plant Physiol. 2005;139(4):1840–52. 10.1104/pp.105.066688 . 16299184 PMC1310563

[12] Piovan A, Filippini R. Anthocyanins in Catharanthus roseus in vivo and in vitro: a review. Phytochemistry Reviews. 2007;6(2–3):235–42. 10.1007/s11101-006-9052-y

[13] Gao J-J, Shen X-F, Zhang Z, Peng R-H, Xiong A-S, Xu J, et al The myb transcription factor MdMYB6 suppresses anthocyanin biosynthesis in transgenic Arabidopsis. Plant Cell, Tissue and Organ Culture (PCTOC). 2011;106(2):235–42. 10.1007/s11240-010-9912-4

[14] Treutter D. Biosynthesis of phenolic compounds and its regulation in apple. Plant Growth Regulation. 2001;34(1):71–89. 10.1023/a:1013378702940

[15] Hong Y, Tang X, Huang H, Zhang Y, Dai S. Transcriptomic analyses reveal species-specific light-induced anthocyanin biosynthesis in chrysanthemum. BMC Genomics. 2015;16(1):202 10.1186/s12864-015-1428-1 . 25887322 PMC4404602

[16] Reif H, Niesbach U, Deumling B, Saedler H. Cloning and analysis of two genes for chalcone synthase from Petunia hybrida. Molecular and General Genetics MGG. 1985;199(2):208–15. 10.1007/BF00330261

[17] Britsch L, DEDIO J, SAEDLER H, FORKMANN G. Molecular characterization of flayanone 3β-hydroxylases. European Journal of Biochemistry. 1993;217(2):745–54. 10.1111/j.1432-1033.1993.tb18301.x 8223617

[18] Jez JM, Bowman ME, Dixon RA, Noel JP. Structure and mechanism of the evolutionarily unique plant enzyme chalcone isomerase. Nature Structural & Molecular Biology. 2000;7(9):786–91. 10.1038/79025 . 10966651

[19] Marles MS, Ray H, Gruber MY. New perspectives on proanthocyanidin biochemistry and molecular regulation. Phytochemistry. 2003;64(2):367–83. 10.1016/S0031-9422(03)00377-7 . 12943753

[20] Fernandes de Oliveira A, Mercenaro L, Del Caro A, Pretti L, Nieddu G. Distinctive Anthocyanin Accumulation Responses to Temperature and Natural UV Radiation of Two Field-Grown (Vitis vinifera L.) Cultivars. Molecules. 2015;20(2):2061–80. 10.3390/molecules20022061 . 25633334 PMC6272526

[21] Ekici L, Simsek Z, Ozturk I, Sagdic O, Yetim H. Effects of Temperature, Time, and pH on the Stability of Anthocyanin Extracts: Prediction of Total Anthocyanin Content Using Nonlinear Models. Food Analytical Methods. 2013;7(6):1328–36. 10.1007/s12161-013-9753-y

[22] Takos AM, Jaffe FW, Jacob SR, Bogs J, Robinson SP, Walker AR. Light-induced expression of a MYB gene regulates anthocyanin biosynthesis in red apples. Plant Physiol. 2006;142(3):1216–32. 10.1104/pp.106.088104 . 17012405 PMC1630764

[23] Liu T, Song S, Yuan Y, Wu D, Chen M, Sun Q, et al Improved peach peel color development by fruit bagging. Enhanced expression of anthocyanin biosynthetic and regulatory genes using white non-woven polypropylene as replacement for yellow paper. Scientia Horticulturae. 2015;184:142–8. 10.1016/j.scienta.2015.01.003

[24] Sharma RR, Reddy SVR, Jhalegar MJ. Pre-harvest fruit bagging: a useful approach for plant protection and improved post-harvest fruit quality—a review. J Horticult Sci Biotechnol. 2014;89(2):101–13. 10.1080/14620316.2014.11513055 R. R. WOS:000335092700001.

[25] Solfanelli C, Poggi A, Loreti E, Alpi A, Perata P. Sucrose-specific induction of the anthocyanin biosynthetic pathway in Arabidopsis. Plant physiology. 2006;140(2):637–46. 10.1104/pp.105.072579 . 16384906 PMC1361330

[26] Jeong S-W, Das PK, Jeoung SC, Song J-Y, Lee HK, Kim Y-K, et al Ethylene suppression of sugar-induced anthocyanin pigmentation in Arabidopsis. Plant physiology. 2010;154(3):1514–31. 10.1104/pp.110.161869 20876338 PMC2971625

[27] Hara M, Oki K, Hoshino K, Kuboi T. Enhancement of anthocyanin biosynthesis by sugar in radish (Raphanus sativus) hypocotyl. Plant Science. 2003;164(2):259–65. 10.1016/S0168-9452(02)00408-9

[28] Van den Ende W, El-Esawe SK. Sucrose signaling pathways leading to fructan and anthocyanin accumulation: A dual function in abiotic and biotic stress responses? Environmental and Experimental Botany. 2014;108:4–13. 10.1016/j.envexpbot.2013.09.017

[29] Lancaster JE, Dougall DK. Regulation of skin color in apples. Critical Reviews in Plant Sciences. 1992;10(6):487–502. 10.1080/07352689209382324

[30] Wang H, Arakawa O, Motomura Y. Influence of maturity and bagging on the relationship between anthocyanin accumulation and phenylalanine ammonia-lyase (PAL) activity in ‘Jonathan’ apples. Postharvest Biology and Technology. 2000;19(2):123–8. 10.1016/S0925-5214(00)00089-2

[31] Perring MA, Clijsters H. The chemical composition and storage characteristics of apples grown in black cloth bags. Plant Food Hum Nutr. 1974;23(4):379–93. 10.1007/BF01095426

[32] Dong Y, Guan J-F, Ma S-J, Liu L-L, Feng Y-X, Cheng Y-D. Calcium content and its correlated distribution with skin browning spot in bagged Huangguan pear. Protoplasma. 2015;252(1):165–71. 10.1007/s00709-014-0665-5 . 24965371

[33] McGuire RG. Reporting of objective color measurements. HortScience. 1992;27(12):1254–5.

[34] Huang C, Yu B, Teng Y, Su J, Shu Q, Cheng Z, et al Effects of fruit bagging on coloring and related physiology, and qualities of red Chinese sand pears during fruit maturation. Scientia Horticulturae. 2009;121(2):149–58. 10.1016/j.scienta.2009.01.031

[35] Arnon DI. COPPER ENZYMES IN ISOLATED CHLOROPLASTS. POLYPHENOLOXIDASE IN BETA VULGARIS. Plant Physiology. 1949;24(1):1–15. . 16654194 10.1104/pp.24.1.1 PMC437905

[36] Liu Y, Che F, Wang L, Meng R, Zhang X, Zhao Z. Fruit Coloration and Anthocyanin Biosynthesis after Bag Removal in Non-Red and Red Apples (*Malus × domestica* Borkh.). Molecules. 2013;18(2):1549–63. 10.3390/molecules18021549 23353125 PMC6269864

[37] Sharma RR, Pal RK, Asrey R, Sagar VR, Dhiman MR, Rana MR. Pre-harvest fruit bagging influences fruit color and quality of apple cv. Delicious. Agricultural Sciences. 2013;Vol.04 No.09:6 10.4236/as.2013.49059

[38] Chen C-S, Zhang D, Wang Y-Q, Li P-M, Ma F-W. Effects of fruit bagging on the contents of phenolic compounds in the peel and flesh of ‘Golden Delicious’, ‘Red Delicious’, and ‘Royal Gala’ apples. Scientia Horticulturae. 2012;142:68–73. 10.1016/j.scienta.2012.05.001

[39] Ju Z. Fruit bagging, a useful method for studying anthocyanin synthesis and gene expression in apples. Scientia Horticulturae. 1998;77(3–4):155–64. 10.1016/S0304-4238(98)00161-7

[40] Moriguchi T, Sanada T, Yamaki S. Seasonal fluctuations of some enzymes relating to sucrose and sorbitol metabolism in peach fruit. Journal of the American Society for Horticultural Science. 1990;115(2):278–81.

[41] Saure MC. External control of anthocyanin formation in apple. Scientia Horticulturae. 1990;42(3):181–218. 10.1016/0304-4238(90)90082-P

[42] Ban Y, Kondo S, Ubi BE, Honda C, Bessho H, Moriguchi T. UDP-sugar biosynthetic pathway: contribution to cyanidin 3-galactoside biosynthesis in apple skin. Planta. 2009;230(5):871–81. 10.1007/s00425-009-0993-4 . 19652996

[43] Iglesias I, Echeverría G, Soria Y. Differences in fruit colour development, anthocyanin content, fruit quality and consumer acceptability of eight ‘Gala’ apple strains. Scientia Horticulturae. 2008;119(1):32–40. 10.1016/j.scienta.2008.07.004

[44] Palapol Y, Ketsa S, Stevenson D, Cooney JM, Allan AC, Ferguson IB. Colour development and quality of mangosteen (Garcinia mangostana L.) fruit during ripening and after harvest. Postharvest Biology and Technology. 2009;51(3):349–53. 10.1016/j.postharvbio.2008.08.003

[45] Usenik V, Stampar F, Veberic R. Anthocyanins and fruit colour in plums (Prunus domestica L.) during ripening. Food Chemistry. 2009;114(2):529–34. 10.1016/j.foodchem.2008.09.083

[46] Li M, Feng F, Cheng L. Expression patterns of genes involved in sugar metabolism and accumulation during apple fruit development. PLoS One. 2012;7(3):e33055 10.1371/journal.pone.0033055 . 22412983 PMC3296772

[47] Noro S, Kudo N, Kitsuwa T. Differences in Sugar and Organic Acid Contents between Bagged and Unbagged Fruits of the Yellow Apple Cultivars, and the Effect on Development of Anthocyanin. Journal of the Japanese Society for Horticultural Science. 1989;58(1):17–24. 10.2503/jjshs.58.17

[48] O’Hara LE, Paul MJ, Wingler A. How Do Sugars Regulate Plant Growth and Development? New Insight into the Role of Trehalose-6-Phosphate. Molecular plant. 2013;6(2):261–74. 10.1093/mp/sss120 . 23100484

[49] Fitzenberger E, Deusing D, Wittkop A, Kler A, Kriesl E, Bonnländer B, et al Effects of Plant Extracts on the Reversal of Glucose-Induced Impairment of Stress-Resistance in Caenorhabditis elegans. Plant Foods Hum Nutr. 2014;69(1):78–84. 10.1007/s11130-013-0399-0 . 24390728

[50] Galant AL, Kaufman RC, Wilson JD. Glucose: Detection and analysis. Food Chemistry. 2015;188:149–60. 10.1016/j.foodchem.2015.04.071 . 26041177

[51] Das PK, Shin DH, Choi SB, Park YI. Sugar-hormone cross-talk in anthocyanin biosynthesis. Mol Cells. 2012;34(6):501–7. 10.1007/s10059-012-0151-x . 22936387 PMC3887831

[52] Baena-González E, Sheen J. Convergent energy and stress signaling. Trends in Plant Science. 2008;13(9):474–82. 10.1016/j.tplants.2008.06.006 . 18701338 PMC3075853

[53] Lopez-Bucio J, Nieto-Jacobo MF, Ramırez-Rodrıguez V, Herrera-Estrella L. Organic acid metabolism in plants: from adaptive physiology to transgenic varieties for cultivation in extreme soils. Plant Science. 2000;160(1):1–13. 10.1016/S0168-9452(00)00347-2 11164572

[54] Chen C, Hu W, He Y, Jiang A, Zhang R. Effect of citric acid combined with UV-C on the quality of fresh-cut apples. Postharvest Biology and Technology. 2016;111:126–31. 10.1016/j.postharvbio.2015.08.005

[55] Chen M, Jiang Q, Yin X-R, Lin Q, Chen J-Y, Allan AC, et al Effect of hot air treatment on organic acid- and sugar-metabolism in Ponkan (Citrus reticulata) fruit. Scientia Horticulturae. 2012;147:118–25. 10.1016/j.scienta.2012.09.011

[56] Park S-H, Choi M-R, Park J-W, Park K-H, Chung M-S, Ryu S, et al Use of Organic Acids to InactivateEscherichia coliO157:H7,SalmonellaTyphimurium, andListeria monocytogeneson Organic Fresh Apples and Lettuce. Journal of Food Science. 2011;76(6):M293–M8. 10.1111/j.1750-3841.2011.02205.x . 21623781

[57] Rahman SME, Jin Y-G, Oh D-H. Combination treatment of alkaline electrolyzed water and citric acid with mild heat to ensure microbial safety, shelf-life and sensory quality of shredded carrots. Food Microbiology. 2011;28(3):484–91. 10.1016/j.fm.2010.10.006 . 21356455

