# Supplementary material for: Effect of Debagging Time on Pigment Patterns in the Peel and Sugar and Organic Acid Contents in the Pulp of ‘Golden Delicious’ and ‘Qinguan’ Apple Fruit at Mid and Late Stages of Development
Source: PLoS One. 2016 Oct 27;11(10):e0165050. doi: 10.1371/journal.pone.0165050 (PMC5082798; doi:10.1371/journal.pone.0165050)
Supplement: S1 Table — B-baging, DB-debagging, H-harvesting. (DOCX) [file pone.0165050.s001.docx]

**S1 Table. The treatments and harvesting time of both ‘Golden delicious’ and ‘Qinguan’ apple fruit.** B-baging, DB-debagging, H-harvesting

| Time | 45DAF | 90/126  (‘Golden delicious’/ ‘Qinguan’) | | 108/137  (‘Golden delicious’/ ‘Qinguan’) | | 122/152  (‘Golden delicious’/ ‘Qinguan’) | | 138/168  (‘Golden delicious’/ ‘Qinguan’) | | 145/179  (‘Golden delicious’/ ‘Qinguan’) | | 152/187  （‘Golden delicious’/ ‘Qinguan’） | | 160/196  （‘Golden delicious’/ ‘Qinguan’） |
| --- | --- | --- | --- | --- | --- | --- | --- | --- | --- | --- | --- | --- | --- | --- |
| CK (no-bagging) | No-bagging | H | H | | H | | H | | H | | H | | H | |
| T1 | B | DB and H | H | | H | | H | | H | | H | | H | |
| T2 | B |  | DB and H | | H | | H | | H | | H | | H | |
| T3 | B |  |  | | DB and H | | H | | H | | H | | H | |
| T4 | B |  |  | |  | | DB and H | | H | | H | | H | |
| T5 | B |  |  | |  | |  | | DB and H | | H | | H | |
| T6 | B |  |  | |  | |  | |  | | DB and H | | H | |
| T7 | B |  |  | |  | |  | |  | |  | | D DB and H | |
