# Supplementary material for: Effect of Debagging Time on Pigment Patterns in the Peel and Sugar and Organic Acid Contents in the Pulp of ‘Golden Delicious’ and ‘Qinguan’ Apple Fruit at Mid and Late Stages of Development
Source: PLoS One. 2016 Oct 27;11(10):e0165050. doi: 10.1371/journal.pone.0165050 (PMC5082798; doi:10.1371/journal.pone.0165050)
Supplement: S2 Table — Fruit was collected from 90 to 160 days after flowering (DAF) (‘Golden Delicious’) and 126 to 196 DAF (‘Qinguan’). CK, no-bagging; T1, debagging at 90/126 DAF (‘Golden Delicious’ / ‘Qinguan’); T2, debagging at 108/137 DAF; T3, debagging at 122/152 DAF; T4, debagging at 138/168 DAF; T5, debagging at 145/179 DAF; T6, debagging at 152/187 DAF; and T7, debagging at 160/196 DAF. Lowercase letters indicate statistically significant differences among treatments at all sample date with 9 independent replicates (P < 0.05). (DOCX) [file pone.0165050.s002.docx]

**S2 Table.** **The statistical results shows significant differences in a* values’ of ‘Golden delicious’ and ‘Qinguan’**.

| a* values | ‘Golden delicious’ | | | | | | | ‘Qinguan’ | | | | | | |
| --- | --- | --- | --- | --- | --- | --- | --- | --- | --- | --- | --- | --- | --- | --- |
| DAF(day) | 91 | 107 | 124 | 135 | 142 | 150 | 159 | 126 | 137 | 152 | 168 | 176 | 185 | 196 |
| CK | n | mn | jkl | jkl | hij | fgh | efg | s | rs | q | no | i | fg | c |
| T1 | mn | mn | lm | kl | ijk | gh | fg | rs | o | l | p | k | h | d |
| T2 |  | lm | kl | kl | ij | de | def |  | qrs | j | n | j | h | b |
| T3 |  |  | ijk | fg | efg | efg | cd |  |  | qr | m | e | ef | abc |
| T4 |  |  |  | hi | cde | b | c |  |  |  | qr | e | de | ab |
| T5 |  |  |  |  | gh | b | b |  |  |  |  | q | g | a |
| T6 |  |  |  |  |  | cd | a |  |  |  |  |  | q | ab |
| T7 |  |  |  |  |  |  | b |  |  |  |  |  |  | p |
