# Supplementary material for: Effect of Debagging Time on Pigment Patterns in the Peel and Sugar and Organic Acid Contents in the Pulp of ‘Golden Delicious’ and ‘Qinguan’ Apple Fruit at Mid and Late Stages of Development
Source: PLoS One. 2016 Oct 27;11(10):e0165050. doi: 10.1371/journal.pone.0165050 (PMC5082798; doi:10.1371/journal.pone.0165050)
Supplement: S5 Table — The harvesting and debagging time were shown in S1 Table. Lowercase letters indicate statistically significant differences among treatments at all sample date with 3 replicates (P < 0.05). (DOCX) [file pone.0165050.s005.docx]

**S5 Table. The statistical results shows significant differences in Malic acid, citric acid and succinic acid of ‘Golden delicious’ and ‘Qinguan’**.

|  |  | ‘Golden delicious’ | | | | | | | ‘Qinguan’ | | | | | | |
| --- | --- | --- | --- | --- | --- | --- | --- | --- | --- | --- | --- | --- | --- | --- | --- |
|  | DAF (day) | 90 | 104 | 116 | 130 | 137 | 145 | 160 | 126 | 137 | 152 | 168 | 179 | 187 | 196 |
| Malic  acid | CK | a | b | b | bc | bc | cde | ghi | ab | d | c | de | def | de | hi |
|  | T1 | b | bc | bcd | def | fg | fgh | kl | c | abc | def | efg | fgh | hi | ij |
|  | T2 |  | cd | efg | fg | fg | fghi | jk |  | c | bc | abc | ijkl | ghi | efg |
|  | T3 |  |  | fgh | ijk | klm | lm | n |  |  | a | efg | hi | e | l |
|  | T4 |  |  |  | hi | hij | lm | n |  |  |  | hi | ef | kl | efg |
|  | T5 |  |  |  |  | ghi | ij | lm |  |  |  |  | gh | ij | jkl |
|  | T6 |  |  |  |  |  | lm | lm |  |  |  |  |  | ij | ijk |
|  | T7 |  |  |  |  |  |  | m |  |  |  |  |  |  | l |
| Citric  acid | CK | a | ab | bc | de | de | h | h | ab | cde | fg | bcde | i | lm | m |
|  | T1 | a | ab | de | de | efg | gh | h | a | def | ab | fg | i | hi | lm |
|  | T2 |  | ab | cde | fgh | de | cd | cde |  | abc | abcd | i | ij | jk | kl |
|  | T3 |  |  | bcd | efg | fg | de | gh |  |  | abcd | ghi | fg | ij | hi |
|  | T4 |  |  |  | bcd | de | efg | def |  |  |  | abcd | bc | jkl | m |
|  | T5 |  |  |  |  | bcd | fg | fgh |  |  |  |  | efg | fgh | ij |
|  | T6 |  |  |  |  |  | bcd | efgh |  |  |  |  |  | i | ijk |
|  | T7 |  |  |  |  |  |  | bcd |  |  |  |  |  |  | ij |
| Succinic  acid | CK | a | ab | fghi | jkl | lmn | o | q | b | bcd | cde | jkl | kl | l | m |
|  | T1 | ab | ab | efgh | ghi | klm | lmn | p | a | cde | a | def | bc | ij | fg |
|  | T2 |  | ab | fgh | ij | ijk | fgh | p |  | a | ghi | cde | hi | efg | hi |
|  | T3 |  |  | abc | ghi | ijk | lm | no |  |  | a | def | efg | kl | jkl |
|  | T4 |  |  |  | bcd | jklm | jkl | ij |  |  |  | a | fgh | l | fg |
|  | T5 |  |  |  |  | cde | klm | klm |  |  |  |  | b | ed | ijk |
|  | T6 |  |  |  |  |  | def | mn |  |  |  |  |  | bcde | hi |
|  | T7 |  |  |  |  |  |  | hi |  |  |  |  |  |  | i |
